# Covalent Modification of Keap1 by the Key Metabolic Cofactor Coenzyme A Under Oxidative and Metabolic Stress

**DOI:** 10.3390/antiox15060702

**Published:** 2026-06-01

**Authors:** Xuezhe Zhou, Oksana Malanchuk, Dejun Zhang, Alexander Zhyvoloup, Maria-Armineh Tossounian, Takafumi Suzuki, Masayuki Yamamoto, Valeriy Filonenko, Jerome Gouge, Ivan Gout

**Affiliations:** 1 Department of Structural and Molecular Biology, University College London, London WC1E 6BT, UK; xuezhe.zhou.20@alumni.ucl.ac.uk (X.Z.); o.malanchuk@ucl.ac.uk (O.M.); dejun.zhang.20@ucl.ac.uk (D.Z.); a.zhyvoloup@ucl.ac.uk (A.Z.); m.tossounian@ucl.ac.uk (M.-A.T.); j.gouge@ucl.ac.uk (J.G.); 2 Department of Cell Signalling, Institute of Molecular Biology and Genetics, National Academy of Sciences of Ukraine, 03680 Kyiv, Ukraine; filonenko@edu.imbg.org.ua; 3 Department of Medical Biochemistry, Graduate School of Medicine, Tohoku University, Sendai 980-8575, Japan; takafumi.suzuki.d5@tohoku.ac.jp (T.S.); masayuki.yamamoto.c7@tohoku.ac.jp (M.Y.)

**Keywords:** Keap1/Nrf2, coenzyme A, post-translational modification, CoAlation, antioxidant response

## Abstract

Kelch-like ECH-associated protein 1 (Keap1) acts as a repressor of nuclear factor-erythroid 2-related factor 2 (Nrf2), a major transcription factor regulating cellular antioxidant response. Keap1 is the substrate adaptor subunit of the cullin 3-RING E3 ubiquitin ligase complex that specifically facilitates Nrf2 ubiquitination and its proteasomal degradation. Keap1 is rich in cysteine residues, and several of them undergo various modifications, such as sulphydration, nitrosylation and glutathionylation under cellular stress conditions. Some of these modifications alter the conformation of Keap1, preventing Nrf2 from ubiquitination and subsequent proteasome-mediated degradation. As a result, newly synthesised Nrf2 translocates to the nucleus to induce the expression of diverse genes involved in protecting cells against oxidative stress. Protein CoAlation is a reversible redox-dependent post-translational modification (PTM) in which coenzyme A (CoA) forms disulphide bonds with oxidised cysteine residues under oxidative or metabolic stress. In this study, we demonstrate for the first time that disulphide Keap1 dimer undergoes CoAlation in cellular response to oxidative stress induced by various oxidising compounds. Furthermore, glucose deprivation also induces CoAlation of the disulphide Keap1 dimer in HEK293/Pank1β cells. We also demonstrate that the Keap1^11 Cys-less^ mutant is not CoAlated in response to diamide treatment or glucose deprivation. In summary, this study uncovers a novel PTM of Keap1 by the key metabolic integrator CoA, which provides new insights into the regulation of the Keap1-Nrf2 antioxidant pathway under oxidative and metabolic stress.

## 1. Introduction

Reduction–oxidation (redox) homeostasis is pivotal for maintaining the biological functions and health of all living cells. Reactive oxygen species (ROS), including hydrogen peroxide (H_2_O_2_) and superoxide anion radical, are generated during aerobic metabolic processes. ROS can also function as critical signalling molecules that modulate diverse biological processes under physiological conditions. However, high levels of ROS deviate redox balance towards oxidative stress that damages biological macromolecules, including proteins, nucleic acids, lipids and carbohydrates. Excessive oxidative stress could lead to ageing and various pathologies, such as cancer, metabolic disorders, neurodegeneration and cardiovascular diseases [1,2].

Intricate cellular defence systems are implicated to counteract oxidative stress, including low-molecular-weight (LMW) thiols and antioxidant enzymes such as catalases, thioredoxins, peroxiredoxins, and redox-sensitive transcription factors [1,2]. LMW thiols, such as glutathione (GSH), bacillithiol (BSH), mycothiol (MSH) and CoA, are a class of compounds characterised by a highly nucleophilic thiol group that can react with ROS for detoxification or protect cellular macromolecules from oxidative or metabolic stress [3,4].

CoA is an indispensable metabolic cofactor generated from cysteine, pantothenic acid and ATP through a highly conserved five-step enzymatic pathway [5]. CoA comprises an ADP moiety linked to a flexible pantetheine tail that ends with a thiol group. Under physiological conditions, CoA forms energy-rich CoA thioesters, which take part in diverse biological processes, such as the tricarboxylic acid (TCA) cycle, the metabolism of amino acids and lipids, signal transduction and regulation of gene expression [5]. Recent findings revealed that in addition to these cellular regulatory processes, CoA acts as an important cellular antioxidant by forming a reversible mixed disulphide bond with protein cysteines (termed protein CoAlation) under oxidative or metabolic stress. Protein CoAlation has been shown to take place in both eukaryotes and prokaryotes exposed to cellular stress. A mass spectrometry-based methodology for the identification of CoAlated proteins has been recently established [5]. More than 2300 proteins have been identified to be targeted by CoAlation [6,7]. CoAlation not only protects protein thiol groups from irreversible oxidation but can also modulate the conformation, subcellular localisation and enzymatic activities of CoAlated proteins [5,8,9,10,11,12,13].

Nrf2 is a major transcription factor coordinating cellular antioxidant events, which upregulates various cytoprotective and antioxidant genes under cellular stress. When translocated to the nucleus, the Neh1 domain of Nrf2 heterodimerises with small musculoaponeurotic fibrosarcoma (sMAF) proteins and then binds to the Cap’n’collar (CNC)-sMaf binding elements (CsMBEs) in the promoters of target genes [14,15,16]. Under physiological conditions, the Neh2 domain of Nrf2 undergoes Keap1-mediated ubiquitination and degradation in the cytoplasm.

Keap1 is the substrate adaptor subunit of the cullin 3 (Cul3)–RING E3 ubiquitin ligase complex that specifically targets Nrf2 for ubiquitination and subsequent proteasomal degradation [17,18,19,20]. Keap1 is a cysteine-rich redox sensor. Several highly reactive cysteine residues in both human and mouse Keap1 were shown to be directly modified by electrophiles and oxidants, including Cys151, Cys273, Cys288, Cys226, Cys613, Cys622 and Cys624 [21,22,23,24,25,26,27,28]. Under stressed conditions, PTMs of Keap1 oxidised cysteine residues impair Nrf2 ubiquitination and subsequent degradation. Consequently, newly synthesised Nrf2 translocates to the nucleus, where it binds to CsMBEs to enhance cellular defence mechanisms against redox imbalance [15].

Keap1 is composed of five domains: the N-terminal domain (NTD), the Broad complex–Tramtrack–Bric-a-brac (BTB) domain, the intervening region (IVR), the Kelch domain and the C-terminal domain (CTD) [15,29]. Keap1 forms a non-covalent homodimer via its BTB domain. Under physiological conditions, Keap1 binds Nrf2 via its Kelch domain. Through its BTB domain, Keap1 then binds to Cul3, which is pre-associated with RING-box protein 1 (RBX1). This assembly forms the active Cul3–RING E3 ubiquitin ligase complex that targets Nrf2 for ubiquitination and proteasome-mediated degradation [18,29]. Oxidative stress induces modifications of Keap1 cysteine residues in some of these domains; specifically, C151 in the BTB domain and C273 and C288 in the IVR domain reduce Keap1-Cul3 E3 ubiquitin ligase activity, while modifications of other cysteine residues could interfere with Keap1-Nrf2 association and ubiquitination [15].

Studies have shown various PTMs on Keap1 cysteine residues, including alkylation, sulphydration, nitrosylation, lactoylation, succinylation, and the formation of methylimidazole (a crosslink between cysteine and arginine). Some of these modifications lead to the activation of the Nrfrf2 pathway [30,31,32,33,34,35,36,37]. Notably, several studies reported Keap1 glutathionylation under oxidative stress, promoting Nrf2 nuclear translocation and upregulation of Nrf2 downstream genes [30,38,39,40,41,42]. Published studies on Keap1 glutathionylation indicate that it is not restricted to monomeric Keap1, as oxidative stress can also modify the disulphide Keap1 dimer in human lung epithelial cells and mouse lung tissues [42]. These findings emphasise the impact of oxidative stress-induced cysteine modifications on Keap1 and subsequent Nrf2 activation, indicating that Keap1 CoAlation may also modulate the antioxidant events mediated by the Keap1-Nrf2 pathway.

The rationale of this study was to examine covalent modification of Keap1 by coenzyme A in cellular response to a panel of oxidising agents and under metabolic stress induced by glucose deprivation. Initially recombinant Keap1 was shown to be CoAlated in vitro, and therefore, we hypothesised that Keap1 may undergo CoAlation in cells under oxidative stress.

## 2. Materials and Methods

### 2.1. Reagents and Chemicals

H_2_O_2_, diamide, rotenone, menadione, *tert*-Butyl hydroperoxide (TBH), N-ethylmaleimide (NEM), β-glycerolphosphate, sodium dodecyl sulphate (SDS), dithiothreitol (DTT) and 1M Tris hydrochloride (Tris-HCl) were obtained from Sigma-Aldrich (Merck Life Science UK Limited, Dorset, UK). The cOmplete™ Protease Inhibitor Cocktail (PIC) EDTA-free was obtained from Roche (#11836170001, Roche Diagnostics GmbH, Mannheim, Germany), and the Precision Plus Protein Dual Colour Standards protein marker was obtained from Bio-Rad (Precision Plus Protein Dual Color Standards, #1610374, Bio-Rad Laboratories, Inc., Watford, UK). The mouse anti-CoA monoclonal antibody (mAb) 1F10 was produced and characterised as reported by Malanchuk et al. [43]. The rabbit anti-HA recombinant antibody (81290-1-RR) polyclonal antibodies were obtained from Proteintech (Proteintech Europe, Manchester, UK). Secondary antibodies Alexa Fluor 680 goat anti-mouse IgG H&L (#A21057) and IRdye 800 CW goat anti-rabbit IgG H&L (#A32735) were obtained from Invitrogen (Thermo Fisher Scientific Inc., Waltham, MA, USA).

### 2.2. Mammalian Cell Culture and Transient Transfection

The human embryonic kidney 293 (HEK293) cell line as well as HEK293 cells stably overexpressing pantothenate kinase 1β (HEK293/Pank1β) were used in cell-based experiments. The construction of HEK293/Pank1β cells was reported in a previous study [5]. The HEK293/Pank1β cell line was generated in our laboratory [5]. HEK293 and HEK293/Pank1β cells were cultured in Dulbecco’s Modified Eagle Medium (DMEM) (Lonza, SLS, Nottingham, UK) containing 10% foetal bovine serum (FBS) (HyClone, SLS, Nottingham, UK), 50 units/mL penicillin and 50 µg/mL streptomycin (Lonza, SLS, Nottingham, UK) and incubated at 37 °C and 5% CO_2_.

The pEF-HA-mKeap1^WT^ and pEF-HA-mKeap1^11Cys-less^ plasmids were kindly provided by Prof. M. Yamamoto. *Escherichia coli* XL-10 Gold competent cells (Agilent Technologies LDA UK Limited, Cheshire, UK) were used for plasmid amplification. Plasmids were purified using the Monarch Spin Plasmid Miniprep Kit (New England Biolabs, Hitchin, UK), following the manufacturer’s protocols.

A total of 6 × 10^5^ million HEK293 and HEK293/Pank1β cells were seeded onto 10 cm tissue culture dishes and transfected at 65% confluence with pEF-HA-mKeap1^WT^ or pEF-HA-mKeap1^11Cys-less^ using the TurboFect transfection reagent (ThermoScientific Dionex, San Jose, CA, USA), following the manufacturer’s protocols.

### 2.3. Cell Treatment with Oxidising Agents and Metabolic Stress

Twenty-four hours after transfection, the complete DMEM medium was replaced with pyruvate-free DMEM containing 5 mM glucose and 10% FBS prior to treatment with oxidising agents. Cells were incubated in this media for a further 24 h to prime them for oxidative stress and were then treated with diamide (20 μM, 100 μM, 250 μM, or 500 μM), H_2_O_2_ (500 μM), menadione (500 μM), rotenone (50 μM), or TBH (1 mM). To induce metabolic stress, the medium was changed 24 h after transfection to pyruvate-free DMEM containing either 5 mM or 0 mM glucose and 10% FBS. Cells subjected to oxidative or metabolic stress were incubated for a further 30 min or 24 h at 37 °C, respectively, and then harvested by gentle pressure washing and centrifuged at 2630× *g* for 5 min at room temperature (RT). The cell pellets were stored at −80 °C.

### 2.4. Cell Lysis and Immunoprecipitation

Cell pellets were resuspended in lysis buffer and incubated on ice for 20 min. The lysis buffer contains 50 mM Tris-HCl pH 7.5, 150 mM NaCl, 5 mM EDTA, 50 mM NaF, 5 mM Na_4_P_2_O_7_ and 1% Triton X-100, with freshly supplemented 100 mM NEM to alkylate unmodified thiol groups, 20 mM β-glycerophosphate and 1× PIC before usage. Total cell lysates (TCLs) were centrifuged at 20,817× *g* for 20 min at 4 °C, and the supernatant was collected for subsequent immunoprecipitation and Western blot (WB) analysis. Protein concentrations were determined by the Pierce Bradford Protein Assay Kit (#23200, ThermoScientific Dionex, San Jose, CA, USA).

The immunoprecipitation of transiently expressed HA-tagged mouse Keap1 wild-type (HA-mKeap1^WT^) protein or a mutant containing 11 substituted cysteine residues (HA-mKeap1^11Cys-less^) from TCLs was performed using 20 μL 100% Protein G Sepharose (Generon, Slough, UK) and 2 μg rabbit anti-HA recombinant antibody per sample. The protein-bound beads were then mixed with an equal volume of 2× SDS loading buffer, boiled and analysed by WB.

### 2.5. Western Blot Analysis

For cell lysates, samples were prepared under non-reducing conditions by mixing with 5× SDS loading buffer without DTT, boiled for 5 min and centrifuged at 16,100× *g* for 1 min.

Immunoprecipitated protein samples were resolved by non-reducing SDS polyacrylamide gel electrophoresis (SDS-PAGE) using a 10% precast gel (Merck Life Science UK Limited, Dorset, UK), and TCLs were resolved by non-reducing SDS-PAGE using a 4–20% precast gel (Merck Life Science UK Limited). SDS-PAGE was performed at 180 V for 60 min. Separated proteins were then transferred to a PVDF membrane (Millipore, Merck Life Science UK Limited, Dorset, UK) using 5× Trans-Blot Turbo Transfer Buffer (#10026938, Bio-Rad Laboratories, Inc., Watford, UK) and a Trans-Blot Turbo transfer system (Bio-Rad Laboratories, Inc., Watford, UK). The membrane was then blocked with Bio-Rad EveryBlot blocking buffer (#12010020, Bio-Rad Laboratories, Inc., Watford, UK) for 10 min at RT. The membrane was incubated with primary antibodies overnight at 4 °C on a shaker. The membrane was then washed with Tris-buffer saline containing 0.1% Tween 20 (TBSt) and then incubated with the secondary antibodies for 30 min at RT. After incubation, the membrane was washed extensively using TBSt, followed by TBS for imaging.

Immunoprecipitated protein samples and TCLs were analysed by WB using anti-CoA (1:6000) and anti-HA (1:5000) antibodies. Primary antibodies were diluted in Bio-Rad EveryBlot blocking buffer. Secondary antibodies were diluted (1:10,000) in the same blocking buffer supplemented with 0.02% SDS.

Fluorescent immunoreactivities were imaged using Odyssey Scanner CLx and Image Studio Lite software version 5.0 (LI-COR Biosciences, LI-COR Biosciences UK Ltd., Cambridge, UK).

### 2.6. Statistical Analysis

The quantitative analysis of Keap1 disulphide dimer CoAlation was performed for two conditions: 500 µM diamide treatment and glucose deprivation.

The densitometry analysis of band intensity from six independent biological replicates for each condition was performed using Image Studio Software, version 6.2 (LI-COR Biosciences, LI-COR Biosciences UK Ltd., Cambridge, UK). CoAlation intensity signal in diamide treatment experiments was normalised against the total HA-Keap1 protein and presented as fold change relative to the untreated sample (0 µM diamide, baseline). In glucose deprivation experiments, the CoAlation intensity signal was normalised to the total HA-Keap1 protein and presented as a fold change relative to normal glucose growth conditions (25 mM glucose, baseline). Data from six independent replicates are presented as the mean ± SEM (Table S1).

Statistical differences were analysed using an unpaired two-tailed *t*-test with Welch’s correction in GraphPad Prism v10.1.0 (GraphPad Software, Inc., Boston, MA, USA); * *p* ≤ 0.05 (at a 95% confidence) were considered statistically significant (*p* ≤ 0.043 for diamide treatment conditions and *p* ≤ 0.0391 for glucose deprivation conditions, respectively) (Figure S2).

## 3. Results

### 3.1. Oxidative Stress Induces CoAlation of HA-mKeap1 in HEK293/Pank1β Cells

We were prompted to investigate Keap1 CoAlation in cells by strong in vitro CoAlation of GST-Keap1. To validate this finding in cells, we used HEK293 cells with stable expression of Pank1β (HEK293/Pank1β), which produce a comparable CoA level to that in rat liver, kidney and primary cardiomyocytes but much higher in comparison with parental HEK293 cells or other established cell lines [5]. The level of CoA in cells/tissues determines the extent of protein CoAlation, and therefore, HEK293/Pank1β cells have been used widely for the investigation of protein CoAlation under oxidative and metabolic stress [5,8,9,10,11,12,13].

**Figure 1:**
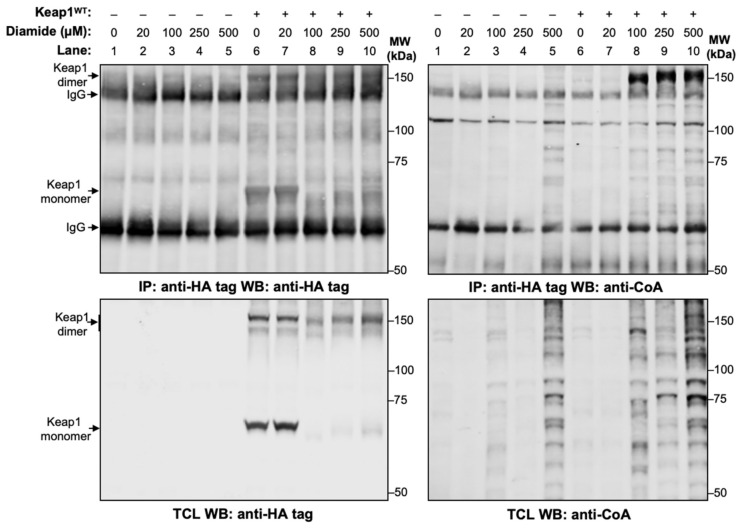
**HA-mKeap1^WT^ is CoAlated in HEK293/Pank1β cells treated with diamide.** HEK293/Pank1β cells were transiently transfected with or without the pEF-HA-mKeap1^WT^ plasmid. After 24 h, the medium was replaced with fresh pyruvate-free DMEM medium containing 5 mM glucose to prime cells for oxidative stress. After culturing for an additional 24 h, cells were treated with or without diamide. TCLs and anti-HA immunoprecipitated samples were separated by SDS-PAGE under the non-reducing condition and analysed by Western blotting with anti-CoA and anti-HA antibodies. The positions of the Keap1 monomer and disulphide dimer and anti-HA antibody (heavy and light chains) are indicated.

Initially, we examined whether transiently overexpressed HA-mKeap1^WT^ is CoAlated in HEK293/Pank1β cells exposed to diamide in a concentration-dependent manner. In this study, HEK293/Pank1β cells were transfected with HA-mKeap1^WT^ and treated with increasing concentrations of diamide or left untreated as the negative control. Anti-HA immunoprecipitated samples from mock-transfected cells were also used as negative controls. As described in Methods, the efficiency of expression and immunoprecipitation of HA-mKeap1^WT^ was confirmed by anti-HA WB of TCL and immunoprecipitated samples (Figure 1). mKeap1 is composed of 624 amino acids with a molecular weight of around 70 kDa [21]. It forms a homodimer (~150 kDa) by non-covalent interactions via the BTB domain in solution [44]. Under non-reducing SDS-PAGE, transiently overexpressed HA-mKeap1^WT^ in untreated cells is predominantly separated as a monomeric form and a less intense covalent dimeric form mediated by disulphide bond formation. Separation of prepared samples under reducing conditions (+DTT) revealed only the monomeric form of HA-Keap1^WT^ in cells, regardless of exposure to diamide or glucose deprivation (Figure S1). The disulphide Keap1 dimer corresponds to two bands at around 150 kDa in TCLs (Figure 1, lanes 6–10). The difference in migration could be attributed to different intramolecular disulphide bond formation within a Keap1 dimer [21]. The intensity of Keap1 monomer and disulphide dimer bands on the anti-HA blot stayed nearly the same in cells treated with 20 μM diamide compared with untreated cells (~2:1 ratio), but the shift from monomer to disulphide dimer was noticeable when cells were treated for 30 min with 100 μM or higher concentrations of diamide (Figure 1, lanes 8–10). Immunoprecipitated HA-mKeap1^WT^ disulphide dimer was found to be strongly CoAlated in a dose-dependent manner, but not the monomer. The intensity of CoAlation increased with higher doses of diamide, corresponding to the decreased mobility of the CoAlated HA-mKeap1^WT^ disulphide dimer. The densitometry analysis indicates that CoAlation of the HA-Keap1 disulphide dimer is greatly induced by oxidative stress caused by treating cells with 500 μM diamide (Table S1, Figure S2). The anti-HA IP and WB of mock-transfected cells showed strong immunoreactivity, corresponding to IgG bands (Figure 1).

**Figure 2:**
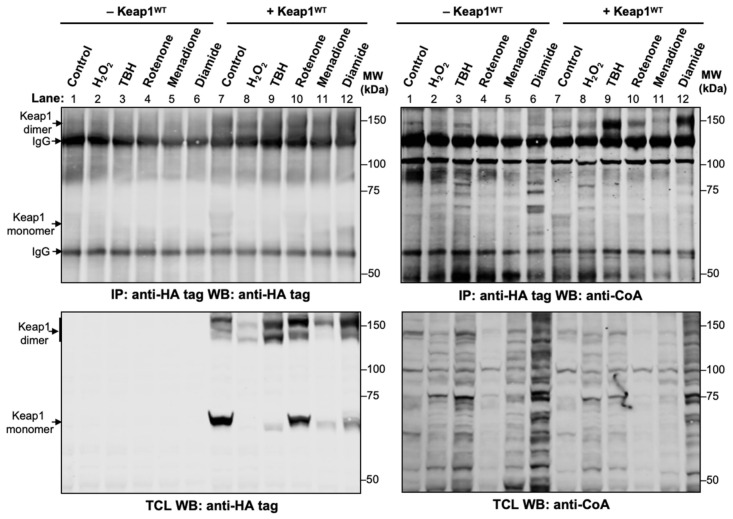
**Induction of Keap1 CoAlation in response to oxidising agents in HEK293/Pank1β cells transiently overexpressing HA-mKeap1^WT^.** HEK293/Pank1β cells were transiently transfected with or without the pEF-HA-mKeap1^WT^ plasmid. After 24 h, the medium was replaced with fresh pyruvate-free DMEM medium containing 5 mM glucose to prime cells for oxidative stress. After culturing for an additional 24 h, cells were treated with or without a panel of oxidising agents. TCLs and anti-HA immunoprecipitated samples were separated by SDS-PAGE under the non-reducing condition and analysed by Western blotting with anti-CoA and anti-HA antibodies. The positions of the Keap1 monomer and disulphide dimer and the anti-HA antibody (heavy and light chains) are indicated.

Diamide induces oxidative stress by perturbing the balance between oxidised and reduced thiols, whereas other oxidising agents function through different mechanisms. H_2_O_2_ and TBH are oxidants while menadione and rotenone increase the production of ROS via redox cycling or disrupting electron transport via complex I, respectively [45,46,47]. After treatment of HEK293/Pank1β cells overexpressing HA-mKeap1^WT^ with different oxidising agents, we observed that diamide and TBH caused the most significant modifications of HA-KEAP1 by CoA and showed a noticeable shift from its monomer to disulphide dimer form on the anti-HA immunoblot (Figure 2, lanes 9 and 12). Cells treated by menadione and H_2_O_2_ showed a significant reduction of HA-mKeap1 monomer in TCLs, while rotenone treatment had a much weaker effect. When the immunoprecipitated samples were immunoblotted with anti-CoA antibody, diamide and TBH treatments showed the strongest CoAlation of the HA-mKeap1 disulphide dimer (Figure 2, lanes 9 and 12). A relatively weak CoAlation of HA-mKeap1 disulphide dimer was observed in rotenone-treated cells (Figure 2, lane 10) but not in cellular response to H_2_O_2_ and menadione when compared to untreated cells (Figure 2, lanes 8 and 11). A weak band of CoAlated HA-mKeap1 in control cells was most likely caused by priming cells (cultured in the pyruvate-free DMEM containing 5 mM glucose overnight, which caused mild metabolic stress) to enhance protein CoAlation under oxidative stress.

### 3.2. Metabolic Stress Induces CoAlation of HA-mKeap1^WT^ in HEK293/Pank1β Cells

**Figure 3:**
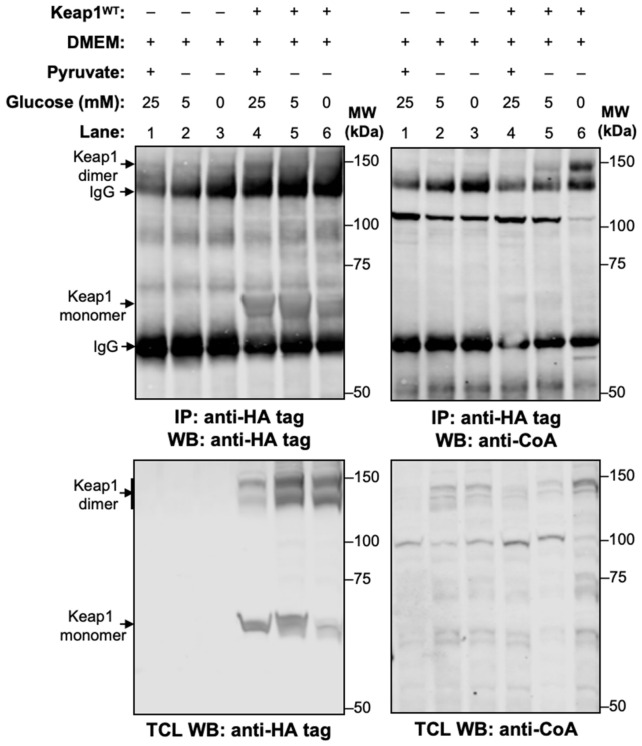
**Metabolic stress induces CoAlation of HA-mKeap1^WT^ in HEK293/Pank1β cells.** HEK293/Pank1β cells were transiently transfected with or without pEF-HA-mKeap1^WT^ plasmid. After 24 h, the medium was replaced with fresh pyruvate-free DMEM medium containing 5 mM glucose or fresh DMEM lacking glucose and pyruvate to induce metabolic stress for 24 h. TCLs and anti-HA immunoprecipitated samples were separated by SDS-PAGE under the non-reducing condition and analysed by Western blotting with anti-CoA and anti-HA antibodies. The positions of the Keap1 monomer and disulphide dimer and the anti-HA antibody (heavy and light chains) are indicated.

In addition to oxidative stress, metabolic stress caused by glucose/pyruvate depletion has been demonstrated to induce protein CoAlation in both mammalian cells and bacteria [5,8]. Therefore, we investigated whether Keap1 is CoAlated in HEK293/Pank1β cells transiently overexpressing HA-mKeap1^WT^ under metabolic stress. The anti-HA immunoblotting of TCLs and anti-HA immunoprecipitated samples under the non-reducing condition revealed that metabolic stress shifted the monomer:disulphide dimer ratio of HA-mKeap1^WT^ towards the dimeric form (Figure 3, lanes 5–6). Probing the same samples with anti-CoA antibody showed that CoAlation of immunoprecipitated HA-mKeap1^WT^ is strongly induced under complete glucose/pyruvate deprivation (Figure 3, lane 6). This increase was clearly detected by densitometry analysis of anti-CoA immunoreactive bands, corresponding to the immunoprecipitated HA-mKeap1^WT^ disulphide dimer (Table S1, Figure S2).

### 3.3. Oxidative Stress Does Not Induce CoAlation of the HA-mKeap1^11Cys-less^ Mutant

**Figure 4:**
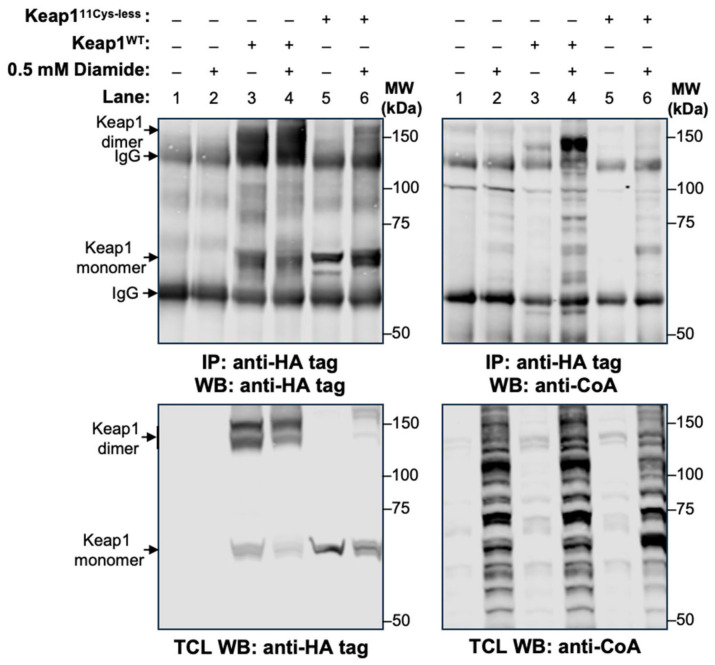
**CoAlation of the HA-mKeap1^11Cys-less^ mutant is not induced by diamide.** HEK293/Pank1β cells were transiently transfected with pEF-HA-mKeap1^WT^ or pEF-HA-mKeap1^11Cys-less^ mutant plasmids. After 24 h, the medium was replaced with fresh pyruvate-free DMEM medium containing 5 mM glucose to prime cells for oxidative stress. After culturing for an additional 24 h, cells were treated with or without diamide. TCLs and anti-HA immunoprecipitated samples were separated by SDS-PAGE under the non-reducing condition and analysed by Western blotting with anti-CoA and anti-HA antibodies. The positions of the Keap1 monomer and disulphide dimer and the anti-HA antibody (heavy and light chains) are indicated.

To further investigate Keap1 CoAlation, HEK293/Pank1β cells were transiently transfected with pEF-HA-mKeap1^WT^ or pEF-HA-mKeap1^11Cys-less^ plasmids, respectively, and then treated with 500 μM diamide. The Keap1^11Cys-less^ mutant lacks 11 cysteine residues that have been implicated in the regulation of the Keap1 function. These include C151, C226, C257, C273, C288, C319, C434, C489, C613, C622 and C624 [28]. The HA-mKeap1^11Cys-less^ was detected as a monomer in untreated cells (Figure 4, lane 5), while diamide treatment caused the appearance of a weak disulphide dimer band on the anti-HA immunoblot (Figure 4, lane 6). The immunoprecipitated HA-mKeap1^WT^ disulphide dimer was strongly CoAlated in response to diamide treatment (Figure 4). However, HA-mKeap1^11Cys-less^ CoAlation was absent after diamide treatment (Figure 4, lane 6), indicating that CoAlation occurs on one or more of these 11 cysteine residues.

## 4. Discussion

In this study, we report for the first time CoAlation as a novel PTM of Keap1 under oxidative and metabolic stress. Covalent modification of Keap1 by CoA expands regulatory mechanisms of the Keap1-Nrf2 pathway and sheds light on the antioxidant role of CoA by modulating a major signalling pathway of oxidative stress response.

It is well known that two Keap1 molecules form a homodimer via non-covalent interactions mediated by the BTB domain under physiological conditions [44,48]. The existence of the Keap1 disulphide dimer has also been reported in cells exposed to oxidative stress. A study using HeLa cells overexpressing mKeap1 demonstrated that the H_2_O_2_-induced Keap1 dimer formation was mediated by a disulphide bond formation between C151 residues of two Keap1 monomers [21]. The formation of a covalently linked Keap1 disulphide dimer was also detected in mouse liver under stressed conditions [49].

**Figure 5:**
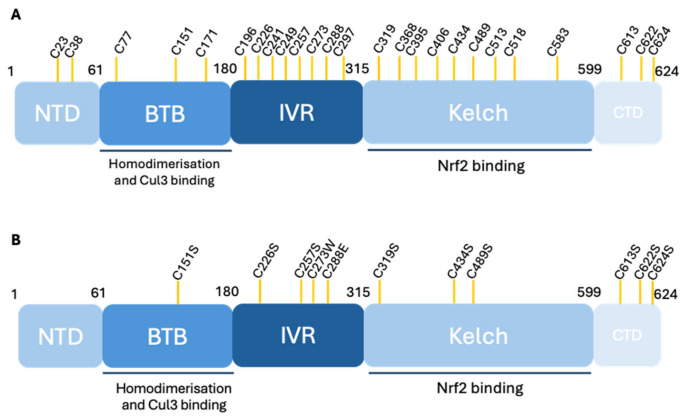
Schematic demonstration of domain organisation of cysteine-rich wild-type mouse Keap1 (**A**) and an 11 Cys-less mutant (**B**).

A central discovery of this study is the CoAlation of the HA-mKeap1^WT^ disulphide dimer in HEK293/Pank1β cells under oxidative stress induced by a range of oxidising agents, including diamide and TBH. A readily detectable CoAlation of HA-Keap1 disulphide dimer was also observed under metabolic stress, induced by glucose deprivation. The densitometry analysis revealed significantly increased CoAlation of the HA-Keap1 disulphide dimer by oxidative stress caused by diamide or glucose deprivation. Notably, CoAlation was not observed in a Keap1 mutant lacking 11 key cysteine residues, which encompass C151 in the BTB domain; C226, C257, C273 and C288 in the IVR domain; C319, C434 and C489 in the Kelch domain; and C613, C622 and C624 in the CTR domain (Figure 5) [28]. We speculate that oxidative and metabolic stress induces the formation of Keap1 disulphide dimers, which promote Keap1 conformational changes, leading to Keap1 CoAlation.

Previous site-directed mutagenesis studies have suggested that C273, C288 and C434 are residues for glutathionylation [41,42]. Mass spectrometry and molecular docking studies demonstrated that C434 can be glutathionylated, which impacts Nrf2 ubiquitination and therefore activates the Nrf2 pathway [42]. An in vitro study has reported that C319 of Keap1 has a high reactivity to form a mixed disulphide bond with GSH [50]. It has been reported that Keap1 C319 is one of the most reactive cysteine residues, which has been reported to be modified by several electrophiles by alkylation to upregulate Nrf2 downstream genes [31,51,52,53,54]. Moreover, it has been shown that C319 can be succinylated by fumarate, contributing to Nrf2 overactivation in fumarate hydratase-related cysts and tumours [33]. Therefore, these four residues could be promising CoAlation sites. Our preliminary data revealed that Keap1 C151S, C273W, C288E, and C434S single mutants were still CoAlated under oxidative stress, indicating that different cysteine residue(s) may be CoAlated.

Under physiological conditions, CoA is mainly involved in intermediary metabolism via the formation of metabolically active thioesters. However, CoA switches to function as a major cellular antioxidant under oxidative or metabolic stress and may mediate stress-induced redox signalling [6,55]. Previous studies have demonstrated that Keap1 is redox-sensitive and can be glutathionylated under oxidative stress in lymphocytes, the brain, pancreatic β cells, lung epithelial cells and several cancer cell lines. Keap1 glutathionylation activates Nrf2-regulated antioxidant and cytoprotective events [30,38,39,40,41,42]. Keap1 CoAlation may cooperate with glutathionylation in facilitating Nrf2-mediated downstream antioxidant responses when cells respond to oxidative or metabolic stress. It has been reported that Nrf2 is activated in skeletal muscles under the fasting condition, which could be related to the Keap1 CoAlation [56].

Several endogenous metabolites have been identified to modify Keap1 and therefore activate Nrf2, including methylglyoxal and glyceraldehyde 3-phosphate that are produced during glycolysis as well as fumarate and itaconate that are derived from the TCA cycle [32,33,34,35]. These findings uncovered the crosstalk between cellular metabolism and stress-response signalling mediated by the Keap1-Nrf2 pathway. Therefore, the discovery of Keap1 CoAlation may highlight a new regulatory mechanism of Keap1 and uncover how a vital metabolic integrator, CoA, functions as a redox effector that connects cellular metabolic states and Nrf2-regulated stress responses. As Keap1 CoAlation is a potential mechanism to protect key cysteine residues from irreversible oxidative damage, Keap1 de-CoAlation to restore its regulatory activity is also worth further investigation [5,57].

The dysregulation of the Keap1-Nrf2 pathway has been implicated in numerous pathologies, including cancer, neurodegenerative disorders and chronic inflammatory diseases [58,59,60]. Our previous study has illustrated extensive anti-CoA immunoreactivity in Alzheimer’s disease post-mortem brain samples as well as tau CoAlation in vitro and in vivo [61]. Thus, understanding how Keap1 CoAlation regulates the Keap1-Nrf2 signalling pathway may facilitate the identification of potential therapeutic targets. Modulating CoA levels could represent an innovative strategy for treating diseases associated with the aberrant Keap1-Nrf2 signalling pathway.

In conclusion, this study adds a novel layer to the complex PTMs of Keap1, demonstrating that CoAlation modifies Keap1 in response to oxidative and metabolic stress. By acting as an antioxidant, CoA transcends its conventional metabolic role and may participate in regulating cellular stress responses through the Keap1-Nrf2 pathway. There are limitations to these original findings. The presented data (which are highly reproducible) rely mainly on the analysis of anti-CoA immunoblotting and immunoprecipitation studies. Furthermore, mass spectrometry and site-specific mutagenesis will be required to determine CoAlated cysteine residue(s) and functional consequences of this modification. In addition, future studies will evaluate the expression levels of Nrf2 under various experimental conditions as well as the analysis of Nrf2 nuclear translocation. The induction of Nrf2 downstream genes, such as heme oxygenase-1 and NAD(P)H:quinone oxidoreductase, will be investigated. In summary, the above studies may offer novel directions for the development of therapeutic interventions that modulate the Keap1-Nrf2 pathway.

## Data Availability

The data that support the findings are included in the article and Supplementary Material. Further enquiries can be directed to the corresponding author.

## Supplementary Materials

The following supporting information can be downloaded at: https://www.mdpi.com/article/10.3390/antiox15060702/s1, Figure S1: Metabolic and oxidative stress-induced covalent dimerisation of transiently overexpressed HA-mKeap1WT in HEK293/Pank1β cells is mediated by a disulphide bond formation; Table S1: The quantitative analysis of HA-Keap1 CoAlation in HEK293/Pank1β cells under oxidative and metabolic stress; Figure S2: Statistical analysis of HA-Keap1 CoAlation in HEK293/Pank1β cells under oxidative and metabolic stress.

## Abbreviations

The following abbreviations are used in this manuscript:

Keap1	Kelch-like ECH-associated protein 1
Nrf2	Nuclear factor-erythroid 2-related factor 2
PTM	Post-translational modification
CoA	Coenzyme A
Redox	Reduction–oxidation
ROS	Reactive oxygen species
H_2_O_2_	Hydrogen peroxide
LMW	Low-molecular-weight
GSH	Glutathione
BSH	Bacillithiol
MSH	Mycothiol
TCA	Tricarboxylic acid
sMAF	Small musculoaponeurotic fibrosarcoma
CNC	Cap’n’collar
CsMBE	CNC-sMaf binding element
Cul3 RBX1	Cullin3 RING-box protein 1
NTD	N-terminal domain
BTB	Broad complex–Tramtrack–Bric-a-brac
IVR	Intervening region
CTD	C-terminal domain
TBH	*tert*-Butyl hydroperoxide
NEM	N-ethylmaleimide
SDS	Sodium dodecyl sulphate
DTT	Dithiothreitol
Tris-HCl	Tris hydrochloride
PIC	Protease Inhibitor Cocktail
mAb	Monoclonal antibody
HEK293	Human embryonic kidney 293
Pank1β	Pantothenate kinase 1β
DMEM	Dulbecco’s Modified Eagle Medium
FBS	Foetal bovine serum
RT	Room temperature
TCL	Total cell lysate
WB	Western blot
HA-mKeap1^WT^	HA-tagged wild-type mouse Keap1
HA-mKeap1^11Cys-less^	HA-tagged mouse Keap1 mutant containing 11 substituted cysteine residues
SDS-PAGE	SDS polyacrylamide gel electrophoresis
TBSt	Tris-buffer saline containing 0.1% Tween 20
