# Supplementary material for: Covalent Modification of Keap1 by the Key Metabolic Cofactor Coenzyme A Under Oxidative and Metabolic Stress
Source: Antioxidants (Basel). 2026 Jun 1;15(6):702. doi: 10.3390/antiox15060702 (PMC13295608; doi:10.3390/antiox15060702)
Supplement: Supplementary file 1 [file antioxidants-15-00702-s001.zip › antioxidants-4291325-supplementary.pdf]

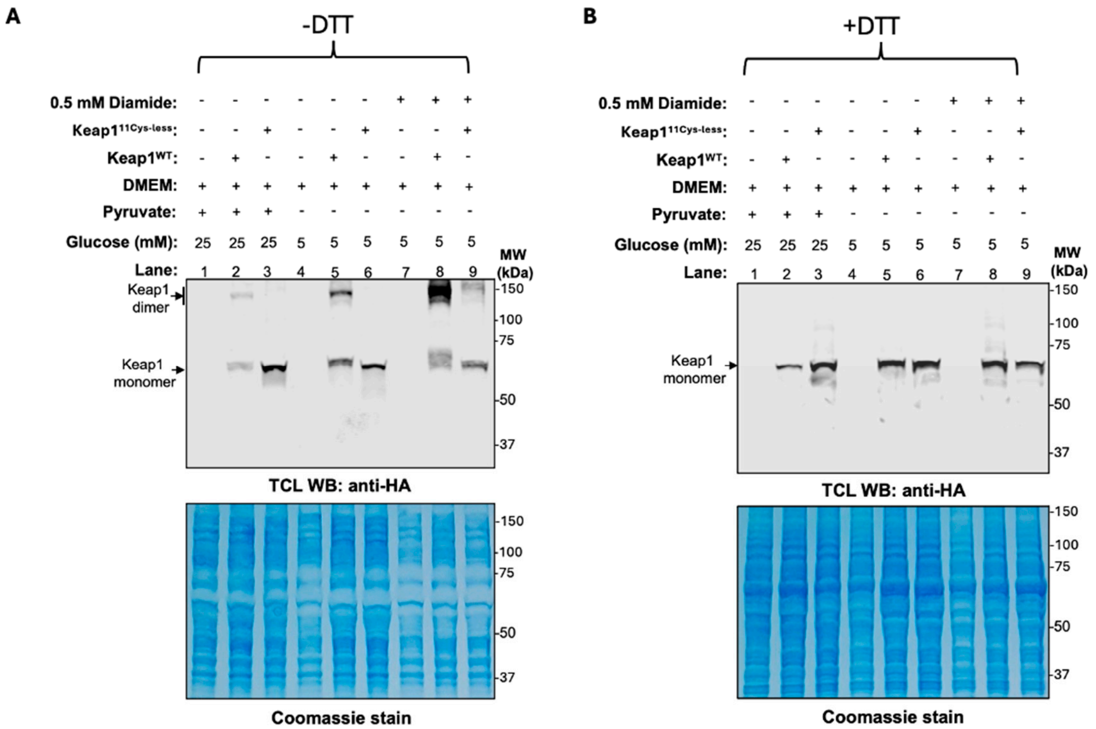

Figure S1. Metabolic and oxidative stress-induced covalent dimerisation of transiently overexpressed HA-mKeap1WT in HEK293/Pank1β cells is mediated by a disulphide bond formation. HEK293/Pank1β cells were transiently transfected with pEF-HA-mKeap1WT or pEF-HA-mKeap111Cys-less mutant plasmids. 24 hours later, the medium was replaced with fresh pyruvate-free DMEM medium containing 5 mM glucose to induce metabolic stress for 24 hours. To induce oxidative stress, cells were treated with or without diamide after culturing for additional 24 hours in the pyruvate-free DMEM medium containing 5 mM glucose. Total cell lysates were separated by SDS-PAGE under the non-reducing condition (A) and the reducing condition (B) and then analysed by Western blotting with the anti-HA antibody. The positions of the Keap1 monomer and disulphide dimer are indicated in the figure. Gels stained by Coomassie blue were shown below the blots.

Experimental setup		AV fold over norm ± SEM
Keap1 WT	500 µM diamide	5.283 ± 1.959
Keap1 WT	0 mM glucose	7.700 ± 2.774

Table S1. The quantitative analysis of HA-Keap1 CoAlation in HEK293/Pank1β cells under oxidative and metabolic stress.

The quantitative analysis of band intensity from six independent replicates for each condition was performed using Image Studio Software version 6.2 (LI-COR Biosciences, Lincoln, NE, USA). CoAlation intensity signal in diamide treatment experiments was normalised against to the total HA-Keap1 protein and presented as fold change relative to the untreated sample (0 µM diamide, baseline). In glucose deprivation experiments, the CoAlation intensity signal was normalised to the total HA-Keap1 protein and presented as fold change relative to normal glucose growth conditions (25 mM glucose). Data from six independent replicates are presented as the mean ± SEM.

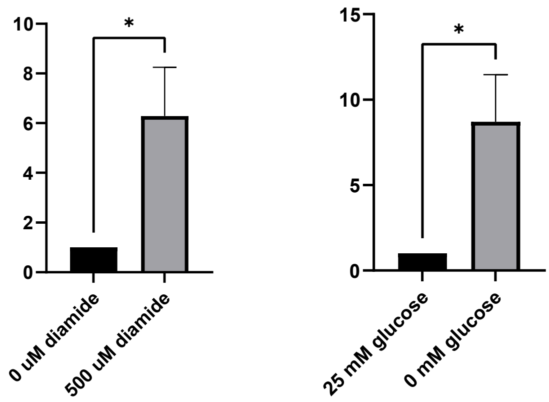

Figure S2. Statistical analysis of HA-Keap1 CoAlation in HEK293/Pank1β cells under oxidative and metabolic stress.

Statistical analysis was performed using an unpaired two-tailed t-test with Welch's correction in GraphPad Prism v10.1.0. Statistical difference is indicated as *$p<0.05$ (at a 95% confidence), confirming statistical significance. Data from six independent replicates.
